# Fatty Acid Profile, Lipid Quality Indices and Oxidative Stability of Snacks Consumed by Children Aged 6–24 Months in Rural Matiari, Sindh, Pakistan

**DOI:** 10.3390/foods14193302

**Published:** 2025-09-24

**Authors:** Shazia Chohan, Sanam I. Soomro, Sarfaraz Ahmed Mahesar, Sheraz Ahmed, Fayaz Umrani, Najeeha T. Iqbal, Junaid Iqbal, Kamran Sadiq, Abdul Khalique Qureshi, Asad Ali, Najma Memon

**Affiliations:** 1 National Centre of Excellence in Analytical Chemistry, University of Sindh, Jamshoro 76080, Pakistan; shaziachemist03@gmail.com (S.C.);; 2 Department of Community Health Sciences, Aga Khan University, Karachi 74800, Pakistan; 3 Department of Biological and Biomedical Sciences, Aga Khan University, Karachi 74800, Pakistan; 4 Department of Paediatrics and Child Health, Aga Khan University, Karachi 74800, Pakistan

**Keywords:** snacks quality, unhealthy foods, Matiari, Pakistan, children’s food, lipid quality indices

## Abstract

High consumption of unhealthy, high-fat snacks negatively affects children’s health, highlighting the need to replace these with healthier alternatives. This study aimed to determine the fatty acid (FA) composition and lipid quality of various branded and local high-fat snacks consumed by children aged 6–24 months in rural Matiari, Sindh. The total energy content of the products ranged from 390.6 to 625.6 kcal/100 g, with fat contributing 9.1 to 47.2 g/100 g. Saturated fatty acids (SFAs) were predominant across samples, particularly palmitic acid (C16:0), ranging from 0.69 ± 0.22 to 16.61 ± 0.1 g/100 g. Among unsaturated fatty acids, oleic acid (C18:1 n-9) was the most prevalent, ranging from 4.63 ± 0.2 to 21.07 ± 0.3 g/100 g. Polyunsaturated fatty acids (PUFAs), especially linoleic acid (C18:2 n-6), were present in lower concentrations. Lipid quality was assessed using four indices: Atherogenic Index (AI), Thrombogenic Index (TI), hypocholesterolemic/hypercholesterolemic (h/H) ratio, and Nutritional Index (NI). Most products exhibited moderate to poor lipid quality, with AI ranging from 0.08 (good) to 1.25 (poor), TI ranging from 0.11 (good) to 1.23 (poor), h/H ratios ranging mostly below 1.0 (undesirable), and NI values ranging from 0.81 to 9.19. In the analyzed snack samples, the results indicate high SFA content, poor lipid quality, and oxidative stability, which may adversely affect children’s health. Changes in dietary habits and the adoption of healthier food choices are strongly recommended to reduce the risk of chronic diseases. Furthermore, understanding the FA profile of foods can support the development of targeted health programs for this population.

## 1. Introduction

The first two years of life represent a critical window period for infants and young children to develop healthy eating behaviors and establish lifelong dietary patterns that support optimal growth and development [1,2]. This period is also marked by heightened vulnerability to growth faltering, micronutrient deficiencies, and common childhood illnesses such as diarrhea and respiratory infections [3]. Undernutrition during these formative years can result in significant short- and long-term consequences, including increased morbidity and mortality, delayed motor development, cognitive impairment, and an elevated risk of non-communicable diseases (NCDs) later in life [4]. Additionally, this phase is crucial for brain development, sensory system maturation, language acquisition, and higher cognitive functioning. The brain undergoes rapid growth during this period, and its development is highly sensitive to both the timing and quality of nutrient intake [5]. Therefore, the age at which complementary foods are introduced, along with their nutritional quality, plays a pivotal role in influencing physical and neurodevelopmental outcomes. Infant and young child feeding (IYCF) practices are not solely about nutrient adequacy—they are also embedded in cultural and social contexts. Mealtimes serve as learning opportunities where children develop food preferences, observe eating behaviors, and form lifelong dietary habits [6,7]. With the dynamic transformation of food environments, there has been a growing prevalence of processed and packaged food products available to all socioeconomic segments.

In Pakistan, high-fat snacks such as biscuits, cakes, chips, and nimko (a fried savory snack mix) are widely consumed, often used as breakfast items or convenient snacks. Biscuits have become a staple snack across the population [8]. The increasing popularity of these snacks among children in rural Matiari, Sindh, is driven by their affordability, palatability, and convenience. In many households, limited cooking facilities and time constraints, especially among mothers who are burdened with household chores and agricultural responsibilities, make it difficult to prepare healthy meals. As a result, these high-fat snacks are often chosen as a quick and easy alternative for feeding children. These products are typically composed of refined flour, sugar, and fats (often hydrogenated or saturated), along with preservatives and flavor enhancers to improve shelf stability and sensory appeal [9,10].

In Pakistan, national food composition tables have limitations concerning basic nutrients and are often not analyzed for these high-fat snack food items, as it is too old, about 15 years; today, eating habits are changing [11]. Previous research is available for only a limited number of chip and biscuit varieties, primarily from selected regions of Punjab and other parts of Pakistan. However, there is a need for broader analysis that considers the dietary patterns of children and adolescents across different age groups, particularly in rural areas where the quality of snacks is generally poorer compared to urban areas. This knowledge gap restricts researchers and policymakers from accurately assessing dietary fat intake and associated health risks. Children aged 6–24 months are particularly vulnerable, as their dietary patterns are being shaped during this critical window period. The early and frequent consumption of high-fat, low-nutrient snacks can displace more nutrient-dense foods, contributing to both undernutrition and increased risk of chronic diseases later in life [12,13]. Given these concerns, this study was undertaken to evaluate the fatty acid composition, lipid quality indices (including Atherogenic Index, Thrombogenic Index, h/H ratio, and Nutritional Index), and oxidative stability of commercially available high-fat snacks commonly consumed by children aged 6–24 months in rural Matiari, Sindh. The findings aim to provide evidence to support the formulation of national dietary recommendations, raise awareness among mothers and caregivers about the potential risks of excessive saturated fat intake, and inform the reformulation of snack products by food manufacturers to enhance their nutritional quality.

## 2. Materials and Methods

### 2.1. Chemicals

All chemicals and reagents used in this experimental design were of analytical grade to ensure analytical precision and reliability. Ethyl acetate and ascorbic acid (Duksan, Ansan, Republic of Korea; copper sulfate, n-heptane, glacial acetic acid, methyl red, methylene blue trihydrate, and isopropanol (Duksan, Ansan, Republic of Korea); sodium thiosulfate pentahydrate, tetrahydrofuran (THF), and hydrochloric acid (Sigma-Aldrich, St. Louis, MO, USA); pyrogallic acid, anhydrous sodium sulfate, and sodium hydroxide (UniChem, Lausanne, Switzerland); hexane, nitric acid (60%), potassium hydroxide, and phenolphthalein (Daejung, Siheung, Republic of Korea); boric acid (Riedel-de Haën, Seelze, Germany); sodium thiosulfate and sulfuric acid (AnalaR Normapur, VWR International, Radnor, PA, USA); boron trifluoride methanol complex, 13–15% BF_3_ (Alfa Aesar, Haverhill, MA, USA); p-anisidine (Fluka, Buchs, Switzerland); potassium iodide (GPR Rectapur, VWR International, Radnor, PA, USA); methyl alcohol (Fisher Scientific, Waltham, MA, USA); potassium bromide and potassium hydroxide (Merck, Darmstadt, Germany); iso-octane and chloroform (LabChem, Zelienople, PA, USA); and ethyl alcohol (AnalAR, VWR Chemicals, Radnor, PA, USA) were used throughout the analytical procedures.

### 2.2. Study Design

The Study of Environmental Enteropathy (SEEM) was conducted between March 2016 and March 2019, with the primary objective of identifying biomarkers of environmental enteropathy in malnourished children. The study enrolled a cohort of 416 children—both malnourished and well-nourished—between 3 and 6 months of age and followed them until 24 months. SEEM was implemented in Matiari, a rural district of Sindh province comprising 18 administrative subunits known as union councils. Each union council has an estimated population of 35,000, and the total population of the district is approximately 0.7 million, with agriculture being the predominant livelihood. The study team has worked extensively in this district since 2003 through various large-scale community-based maternal, neonatal, and child health trials.

This community-based interventional study received ethical approval from the Aga Khan University Ethics Review Committee (ERC #3836-Ped-ERC-15) in December 2015 and is registered on ClinicalTrials.gov (ID: NCT03588013). As part of the SEEM study, detailed dietary data were collected bi-monthly from enrolled children between 6 and 24 months of age using a structured 24 h dietary recall tool. This tool provided quantitative estimates of non-breastmilk food intake, including both home-prepared meals and processed food products.

### 2.3. Dietary Data Collection

The 24 h dietary recall approach was designed to capture all foods and beverages consumed by the child in the previous day. Data were collected using standardized methods that involved direct household observations, weighing of ingredients, and development of household-specific recipes. To support accurate recall, field staff utilized a toolkit comprising measuring utensils, plastic models of fruits and vegetables, food pictorial charts, and an electronic weighing scale for precise gram measurements. Details of the methodology are given in our previously published paper [14]. From this dataset, information regarding the consumption of snack foods—particularly high-fat, processed snacks—was extracted. These snacks included locally and commercially available products such as candies, sweets, cereal bars, chocolates, chips, biscuits, crackers, and nimko (a local mixed snack made from fried pulses, legumes, and nuts). These items were of particular interest due to their widespread consumption and potential implications for child nutrition.

### 2.4. Sampling and Categorization of Snack Foods

Following the completion of dietary data collection for the SEEM Project, snack food sampling was carried out in 2019 in two union councils of Matiari. Product selection was guided by consumption patterns identified through the SEEM 24 h dietary recall data. During sampling, some challenges arose due to the unavailability of certain products, largely attributed to changes in local market dynamics over the 2–3-year gap between dietary assessment and sample collection. Out of 110 high-fat snack items reported, including 49 biscuits, 14 cakes, 37 crisps, and 10 nimko varieties, 44 products were available and collected for laboratory analysis.

All collected samples were categorized into four main groups: biscuits, chips, cakes, and nimko. These groups were further sub-classified based on key ingredient composition by following FAO/INFOODS food matching criteria [15]. The 44 high-fat snack products analyzed in this study were categorized into four main groups based on type and composition. Biscuits (n = 24) were further classified into nine subtypes: whole wheat biscuits, cream sandwich biscuits, sugar-coated biscuits, wafers, zeera biscuits, peanut biscuits, chocolate chip biscuits, and crackers. Chips (n = 8) were categorized according to their primary ingredients, including potato crisps, wheat flour fries, corn flour fries, and mixed wheat flour–potato starch fries. Nimko (n = 6) encompassed products such as nimko mix, dal moth, and locally prepared variants of nimko. Cakes (n = 6), although not grouped due to formulation differences, were individually identified as sponge cake, round cake, fruit cake, cream-filled cupcake, and sandwich cake with cream or chocolate filling.

All samples were homogenized and stored in screw-cap containers at a freezing temperature of 5 °C. Prior to any analysis, samples were thawed and blended at room temperature to ensure uniformity.

Although some local items were no longer obtainable, the 44 selected samples—representing nearly 50% of the originally reported items—were carefully chosen to reflect the diversity of snack types and subtypes, ensuring adequate representation of commonly consumed high-fat snack foods in the study area. This subset was systematically selected based on consumption frequency and food matching criteria, providing comprehensive coverage of all major snack categories identified in the dietary recall data. Composite samples were prepared to capture the full range of ingredient compositions. Therefore, this representative subset offers robust and reliable insights into the fatty acid composition, lipid quality, and oxidative stability of high-fat snacks commonly consumed by children in rural Matiari.

### 2.5. Fat and Fatty Acids Analysis

Fat was extracted using the Soxhlet extraction method, following standard protocols such as the AOCS method Aa 4-38, as described in the ASEAN manual [16]. It proceeds for about 6 h, and hexane was used as a solvent for the extraction process. The solvent was evaporated with a rotatory evaporator at a temperature of 60 °C.

Derivatization of fatty acid methyl esters was performed according to the AOAC method (963.22F). To begin, 0.1 g of oil was weighed in a test tube. A 4 mL solution of NaOH in methanol, with a concentration of 0.5 N, was added and thoroughly mixed. Subsequently, the tube was placed in a water bath and maintained at a temperature range of 85–100 °C for a duration of 5–10 min. Afterward, the solution was allowed to cool, and a 1–5 mL solution of BF3 was vigorously mixed using a vortex. The tube was once again heated at approximately 85 to 100 °C for 15 min, followed by cooling of the solution. Next, 3 mL of heptane and NaCl was added, and the mixture was vigorously shaken. Then the mixture was allowed to settle until the two layers were separated. The upper layer was then carefully transferred, followed by two additional extractions with 2 mL of heptane each time. The resulting solution was passed through a small quantity of anhydrous Na_2_SO_4_ to remove traces of water. Finally, the sample was subjected to analysis using GC/MS, adhering to the provided specifications.

Fatty acid profiling was carried out using an GC–MS instrument (Agilent Technologies, Santa Clara, CA, USA) with Chem-Station (version D.02.00) Scale Mode software. The GC–MS chromatograms found were matched with the NIST library (which identified different types of fatty acid methyl ester present in snack oil). FAMEs were analyzed using agas chromatography (Agilent 6890 N, Agilent Technologies, Santa Clara, CA, USA) instrument connected with an Agilent MS-5975 (Agilent Technologies, Santa Clara, CA, USA). The detector was an inert XL mass selective with an auto sampler (Agilent 7683-B, Agilent Technologies, Santa Clara, CA, USA), and the injector was from Agilent Technologies, Little Falls, NY, USA. The column for FAME separation was a capillary column having model number Agilent 19091S-433 HP-5MS (5% phenyl methyl siloxane) with a length of 30.0 m, a diameter of 250 µm, and a film thickness of 0.25 µm (Agilent Technologies, Palo Alto, CA, USA). The oven temperature program was an initial temperature of 130 °C (held for 1 min), increased by 4 °C/min to 220 °C, then by 10 °C/min to 290 °C, before being held for 3 min, giving a total run time of 33.5 min. Helium (GC-grade, ≥99.999%; Pakistan Oxygen Limited (POL), Karachi, Pakistan) was used as the carrier gas at a constant flow rate of 1.3 mL/min with a column head pressure of 15.98 psi. The injector was operated in split mode (50:1) with an injection volume of 1 µL (10 µL syringe), and injector and detector temperatures were maintained at 300 °C and 290 °C, respectively.

The mass spectrometer was operated with an electron impact (EI) ionization source at 70 eV, an ion source temperature of 230 °C, a quadrupole temperature of 150 °C, and a transfer line temperature of 270 °C. Data were acquired over a mass scan range of 50–550 m/z, with an electron multiplier voltage of 1035 V [15,16].

Calculation of fatty acid methyl esters composition: The percentage area of individual FAME was calculated from the ratio between the area of a single FAME and the sum of the area under all peaks of individual FAME. It was assumed that the ratio of peaks of area is nearly like the mass of the individual FAME. For the calculation of total MUFA, SFA, and PUFA, all individuals’ FAMES were added, and relative percentages were calculated by following the ISO 2014c method [17]. Next, the percentage of fatty acids having been converted to a g/100 gm edible portion using the conversion factor and total fat is shown in the equation below.

Individual FA g/100 gm = (Individual FA%/100) × (XFA × Total fatty acid g/100 gm)

### 2.6. Trans Fat Analysis by FT-IR Spectroscopy

The IR spectra of oils extracted from snack samples of oil were obtained using FT-IR (Thermo Nicolet iS10, Thermo Fisher Scientific, Waltham, MA, USA) with deuterated triglycine sulfate as a detector (DTGS). OMINIC software (versions 9) was used to collect data via functional groups. A background spectrum was obtained before recording the sample spectra of snack oil to minimize interference from air or residues from previous samples. Trans fat was studied qualitatively in the range of 990–945 cm^−1^.

### 2.7. The Lipid Quality Indices

- Index of Atherogenicity (AI):
  AI = (C12:0 + (4 × C14:0) + C16:0)/(Σn-3 PUFA + Σn-6 PUFA + Σ MUFA)
- Index of Thrombogenicity (TI):
  TI = (C14:0 + C16:0 + C18:0)/((0.5 × C18:1) + (0.5 × other MUFA) + (0.5 × Σn-6 PUFA)
  + (3 × Σn-3 PUFA) + Σn-3 PUFA/Σn-6PUFA)
- Hypocholesterolemic Fatty Acids (DFAs):
  DFA = UFA + C18:0
- Hypercholesterolemic Fatty Acids (OFAs):
  OFA = C12:0 + C14:0 + C16:0
- Hypocholesterolemic and Hypercholesterolemic Fatty Acids (H/H):
  H/H = (C18:1n-9 + C18:2n-6 + C18:3n-3)/(C12:0 + C14:0 + C16:0)

All of these calculation formulas were obtained from the study performed by Beata Paszczyk et al., 2020 [18].

f. Nutritional Index (NI)
  NI = ∑(Hypercholesterolemic Fatty Acids)/∑(Hypocholesterolemic Fatty Acids)

where

- Hypocholesterolemic Fatty Acids (h): C18:1 (oleic), C18:2 (linoleic), C18:3 (linolenic);
- Hypercholesterolemic Fatty Acids (H): C14:0 (myristic), C16:0 (palmitic).

The calculation formula was taken from the study performed by Ulbricht, T. L. V. & Southgate, D. A. T. (1991) [19].

### 2.8. Oxidative Stability of Fat Present in High-Fat Snacks:

The p-anisidine (p-AnV), total oxidation (TOTOX), conjugated diene (CD), conjugated triene (CT), free fatty acid (FFA), peroxide value (PV), and fatty acid profiling using GC/MS were carried out by following the standard AOCS, AOAC, and IUPAC methods.

Free fatty acid (FFA) in the oil extracted from snacks was analyzed by following the certified AOCS procedure (Ca 5a-40) using the titrimetric method of analysis. Peroxide values of oil extracted from snack samples were determined using the official AOCS method Cd (8-53) [20]. Iodometric titration was carried out. PV was determined in mEq O_2_/kg of oil samples. The p-AnV of oil extracted from snacks was evaluated using the official AOCS method (Cd 8-53) [21]. The absorbance was measured with the help of UV–Visible spectroscopy. The total oxidative value of the oil extracted from snack samples was calculated as the sum of the peroxide value (PV) and the p-anisidine value (p-AnV). The values of conjugated diene and conjugated triene were determined using the (IUPAC, 1979) official analytical method [22,23,24]. The absorbance at 232 nm for diene and 270 nm for triene was measured in a (carry 100) spectrophotometer, whereas isooctane was used as the blank solvent.

## 3. Results

### 3.1. Energy and Fat Content of High-Fat Snacks:

**Figure 1:**
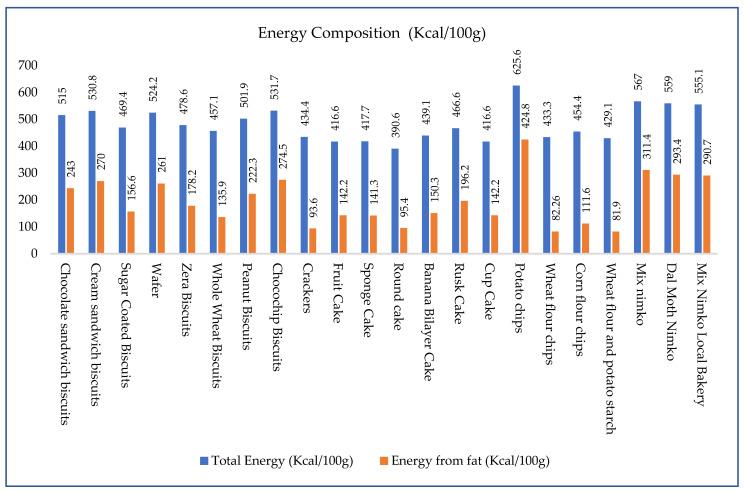
Energy composition (kcal/100 g) of high-fat snacks, highlighting total and fat-derived contributions.

The results in Figure 1 show that the total energy content of high-fat snack products ranged from 390.6 to 625.6 kcal/100 g, with potato chips (625.6 kcal), mix nimko (559–567 kcal), and cream or chocochip biscuits (530.8–531.7 kcal) at the highest levels. These energy-dense foods, despite being consumed in small portions, provide a significant calorie load and can substantially contribute to total daily energy intake, especially among children of 6–24 months, but these calories mostly come from fat and carbohydrates, which are not considered to be very healthy. According to dietary guidelines, children aged 6–24 months require about 930–1140 kcal/day [25], and a typical 50 g serving of potato chips alone delivers over 312 kcal, accounting for nearly 20–25% of a young child’s daily energy requirement; this is often consumed unmonitored between meals or alongside sugary beverages and sweets.

**Figure 2:**
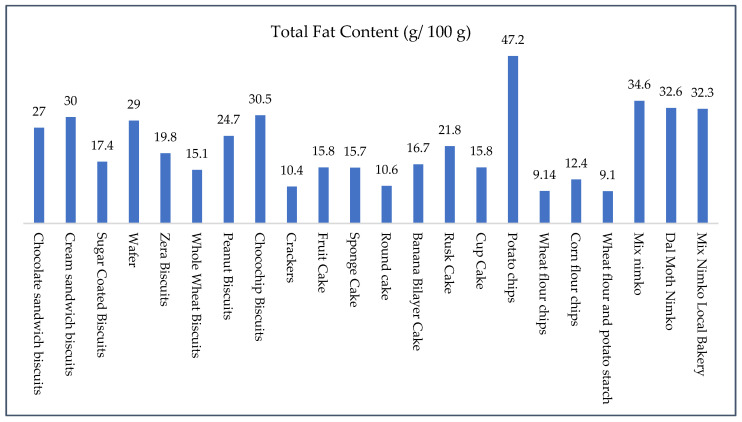
Total fat content (g/100 g) in high-fat snacks.

The energy that comes from fat in Figure 2 shows that products like potato chips (424.8 kcal), mix nimko (311.4 kcal), dal moth nimko (293.4 kcal), and chocochip or cream biscuits (274–270 kcal) derive over 50% of their total energy from fat. This indicates heavy use of oils and hydrogenated fats, which are often rich in saturated and trans fats and are linked to metabolic disturbances in children.

In contrast, products like crackers (93.6 kcal) and wheat-based chips (82–82.26 kcal) had relatively lower energy from fat. However, even these can lead to excessive fat intake when consumed frequently or in multiple servings. Cakes showed slightly lower total energy and fat energy values compared to chips and nimko but still pose nutritional risks when portion sizes are not managed. For instance, fruit cakes, sponge cakes, and cupcakes contribute about 140–150 kcal from fat, which is more than one-third of their total energy. Children are naturally drawn to foods that are crispy, sweet, or salty, being especially pronounced in potato chips, nimko, and biscuits. Repeated exposure and marketing amplify these preferences, often leading to poor dietary habits. Since health authorities recommend that no more than 30–35% of daily energy should come from fat, products contributing 250–300 kcal from fat per 100 g already exceed this threshold in typical serving sizes. For example, a child consuming a 60 g pack of cream biscuits would take in over 318 kcal, including more than 160 kcal from fat, thereby surpassing 50% of the recommended daily fat energy intake in a single snack.

If we look at the fat content of these snacks, these vary widely, from 9.1 g to 47.2 g per 100 g. Potato chips had the highest fat content at 47.2 g, followed by mix nimko (32.3–34.6 g) and cream or chocochip biscuits (30–30.5 g). These levels are alarming given that the RDI for fat in children aged 6–24 years is approximately 30–40/day, depending on total energy needs [25]. Just 50 g of potato chips can provide over 23 g of fat, easily meeting half the daily fat requirement. While slightly lower in fat, products like chocolate sandwich biscuits (27 g), peanut biscuits (24.7 g), and zera biscuits (19.8 g) still contribute significantly to total fat intake, especially when consumed frequently. Even snacks perceived as “healthier”, such as crackers (10.4 g) and whole wheat biscuits (15.1 g), can cumulatively raise fat intake if consumed repeatedly. Among cakes, rusk (21.8 g) and banana bilayer cake (16.7 g) are higher in fat compared to sponge cake (15.7 g) and cupcakes (15.8 g). Chips and nimko showed the widest variability in fat content. Dal moth (32.6 g) and nimko from both branded and local bakeries (32.3–34.6 g) were particularly high in fat. Even corn flour chips (12.4 g) and wheat/potato chips (9.1–9.14 g)—though lower—can contribute significantly to fat intake when portion sizes are large or consumption is habitual. The fat was further analyzed through profiling to determine whether it is healthy or unhealthy, as total fat alone is not enough to make this judgment. Even if the fat content is low, it could still be of poor quality, which is worse. Therefore, we analyzed the fatty acid composition and discussed it to make informed recommendations.

### 3.2. Fatty Acid Composition of High-Fat Snacks

The analysis of fatty acid composition in Table 1 of high-fat snacks revealed that certain fatty acids exceeded recommended levels. Nonetheless, all data were utilized to calculate composite parameters such as the sums of monounsaturated fatty acids (MUFAs), polyunsaturated fatty acids (PUFAs), saturated fatty acids (SFAs), and trans fatty acids (TFAs). Saturated fatty acids were the most predominant fatty acids, followed by fats overall. This may indicate an industry effort to reduce the use of certain harmful fats in response to concerns about their adverse health effects [26]. However, SFAs may be replaced by vegetable fats like palm oil, which is rich in palmitic acid and thus high in SFAs. The elevated SFA levels found in the present high-fat snacks support this possibility. The results suggest that substituting partially hydrogenated fats with palm oil can raise both SFA and TFA content in foods, both of which pose potential health risks. Our data provide quantitative values for fatty acids alongside qualitative detection of trans fats. Palmitic acid (C16:0) was the dominant saturated fatty acid in all samples, ranging from 0.69 g/100 g in round cake to a high of 16.61 g/100 g in potato chips. Excessive intake of palmitic acid from industrial snacks may increase the risk of obesity, inflammation, and poor lipid metabolism later in life. Among MUFAs, oleic acid (C18:1) was predominant, with concentrations ranging from 4.63 g/100 g in crackers to 21.07 g/100 g in potato chips. Palmitoleic acid (C16:1) was either minimal or undetectable in most samples, appearing only in trace amounts, such as 0.06 g/100 g in sponge and round cakes. The consistently high levels of oleic acid (C18:1n9C) across samples likely play a beneficial role in children’s diets by supporting energy metabolism and cardiovascular health. Geraldo and Alfenas (2008) [27] noted that a high-fat diet rich in oleic acid does not provoke the inflammatory response observed with diets high in SFAs and TFAs.

Lauric (C12:0) and palmitic acids have atherogenic effects in humans [28]. It is reported that consumption of oleic, palmitic, and lauric acids was inversely associated with acute myocardial infarction, while no associations were found with myristic (C14:0) and stearic acids [29]. Myristic acid was present in smaller amounts, up to 1.25 g/100 g in chocochip biscuits, while lauric acid was generally absent except for 2.27 g/100 g in chocochip biscuits. Stearic acid (C18:0) showed moderate levels, ranging from 0.29 g/100 g in round cake to 2.26 g/100 g in potato chips. The total saturated fatty acid content (ΣSFA) varied widely, with chocochip biscuits containing the highest amount (17.08 g/100 g) and round cake the lowest (1.11 g/100 g). The PUFA content in all analyzed snack products was considerably lower than the recommended daily intake of 15 g. Wafers contributed only (0.60 g/100 g) 2%, while potato chips contributed about (4.56 g/100 g) 15% of the daily PUFA requirement per 50 g packet serving. These values indicate that, despite their high total fat content, the snacks analyzed are poor sources of essential polyunsaturated fatty acids. This finding suggests the predominant use of oils low in polyunsaturated fatty acids, particularly hydrogenated fats or palm oil, which are known for their inferior nutritional quality. Therefore, replacing these with PUFA-rich oils like sunflower or soybean oil could significantly improve the nutritional quality of these snacks. An exception was round cakes, which matched sunflower oil profiles. Chocochip biscuits contained lauric acid (7.83%) and myristic acid (4.29%), suggesting a blend of oils. The fats and oils used in these snacks vary in their content of SFAs, MUFAs, and PUFAs. The predominant SFAs—palmitic and stearic acids—are associated with increased LDL cholesterol, cardiovascular risk, and diabetes mellitus [30,31,32].

**Figure 3:**
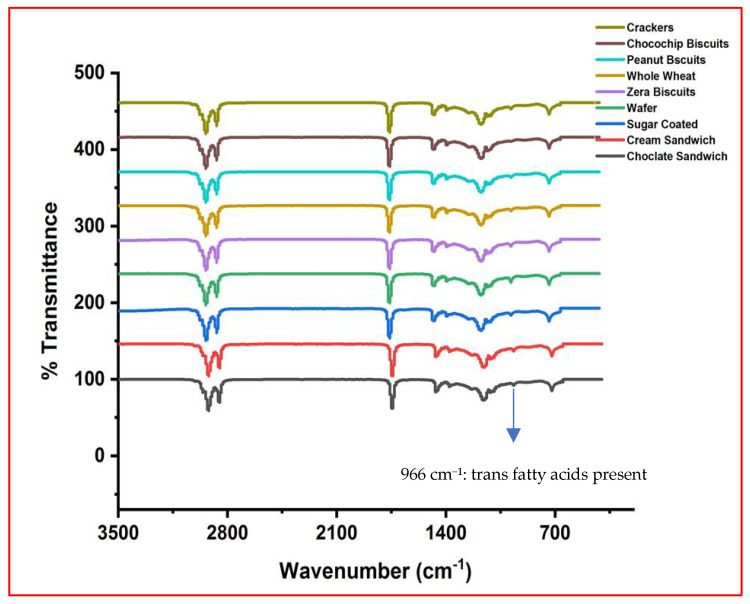
FTIR spectrum of high-fat snack samples—biscuits category.

**Figure 4:**
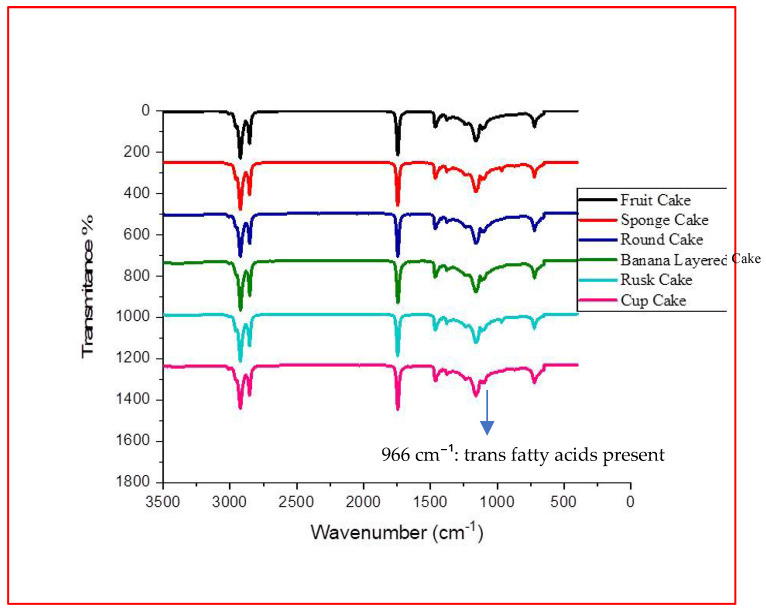
FTIR spectrum of high-fat snack samples—cake category.

**Figure 5:**
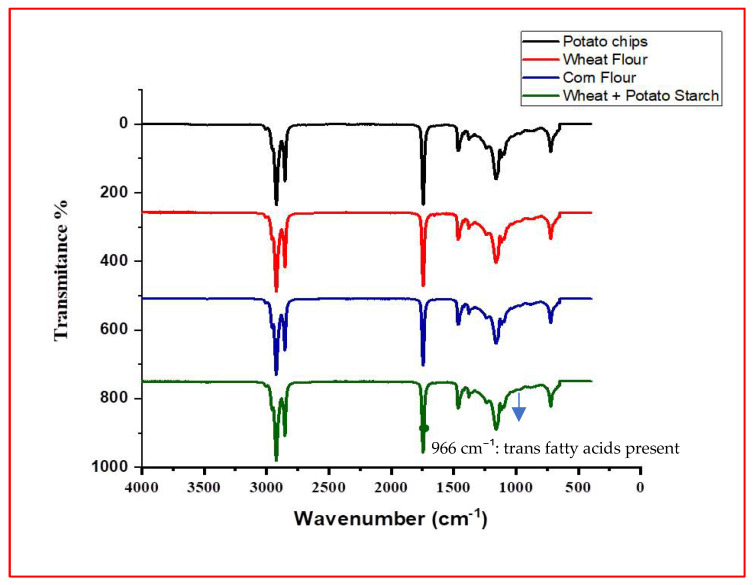
FTIR spectrum of high-fat snack samples—chip category.

**Figure 6:**
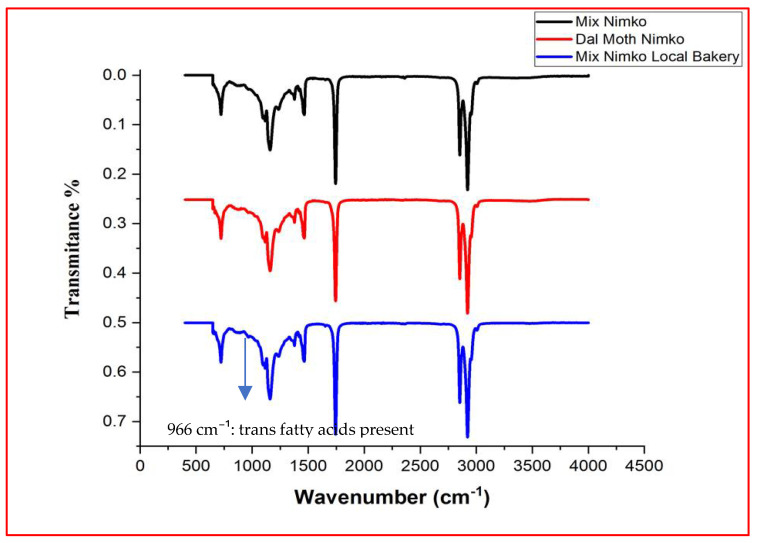
FTIR spectrum of high-fat snack samples—nimko category.

Although quantitative determination of trans fatty acids (TFAs) was not possible in the present study due to limited access to analytical facilities, their presence was qualitatively verified through FTIR, as indicated by the characteristic absorption peak at 966 cm^−1^ in all spectra (Figure 3, Figure 4, Figure 5 and Figure 6). Previous investigations on Pakistani biscuits and chips have reported TFA levels exceeding 5% of the total oil content, which is above the recommended limit. Based on these findings, it can be inferred that the products analyzed in the present study may also contain trans fats at levels higher than the recommended threshold, whereas the World Health Organization (WHO) recommends limiting TFAs to less than 1% of total energy intake [33,34]. Since 2004, Denmark has implemented a legal limit of 2% TFAs in oils and fats used in processed foods [35,36]. Many developed countries have mandated the declaration of TFA content on nutritional labels. However, such regulations are not enforced in local food sectors in Pakistan, where high-fat snacks are often prepared informally and mimic commercial brands without ingredient transparency. Notably, snack serving sizes commonly exceed 100 g/day and are frequently consumed with other high-fat snacks or desserts. This pattern may contribute to excessive intake of saturated fats (SFAs) and TFAs, posing health risks for children. In 2013, the U.S. FDA declared that TFAs are no longer ‘generally recognized as safe’ and initiated regulatory action to eliminate them from the food supply [37,38].

These snacks, rich in SFAs and TFAs but low in essential PUFAs, reflect global convenience food consumption trends that contribute to cardiovascular disease risk—a major public health concern in Pakistan, where non-communicable diseases account for 58% of deaths. This underscores the urgent need for public health strategies emphasizing nutrition education, improved food labeling, and promotion of nutrient-dense diets to foster healthier eating habits from early childhood and reduce long-term health risks. The growing consumption of convenience foods is a global public health issue. Understanding the fatty acid profiles of these foods is essential for designing interventions to prevent chronic diseases. Consequently, the findings of this study have been incorporated into a healthy eating booklet distributed to students. In this study, it was found that a link exists between palmitic acid levels and increased expression of C-reactive protein in aortic endothelial cells, which may contribute to endothelial dysfunction [39].

### 3.3. Lipid Quality Index Assessment

Dietary fat quality is critical for children (6–24 months), as it influences growth, brain development, and the supply of essential fatty acids necessary for optimal health. While total fat intake is important, the type and balance of fatty acids—saturated, monounsaturated, and polyunsaturated—determine the nutritional adequacy of complementary foods. Lipid quality indices, including the Atherogenic Index (AI), Thrombogenic Index (TI), hypocholesterolemic/hypercholesterolemic ratio (h/H), Nutritional Index (NI), and levels of desirable fatty acids (DFAs) and undesirable fatty acids (OFAs), provide a quantitative means to evaluate the fatty acid quality of high-fat snack products for children.

Since no standardized lipid quality index thresholds exist for infants and young children, typical human breast milk values were used as a natural reference for optimal fat intake (AI: ~0.3–0.4, TI: ~0.4–0.5, h/H: ~2.0–2.5, NI: ~2–3, DFA: moderate, OFA: low). The lipid quality of 22 high-fat snacks was assessed against these breast milk benchmarks to provide an approximate evaluation of their suitability as complementary foods during the critical 6–24 months period, shown in Table 2.

Chocochip biscuits had the highest AI (1.25) and TI (1.23), far exceeding typical breast milk values (AI ~ 0.3–0.4, TI ~ 0.4–0.5), which is consistent with their high levels of saturated fatty acids like lauric (C12:0), myristic (C14:0), and palmitic acid (C16:0), all associated with increased LDL cholesterol and atherogenesis. In contrast, round cake and cupcake showed notably low AI (0.08 and 0.28) and TI (0.11 and 0.35), closer to breast milk standards, reflecting healthier fat sources and formulations with lower saturated fat content. The h/H ratio was generally very low (<0.30) across most products, suggesting limited cholesterol-lowering capacity relative to breast milk (h/H ~ 2.0–2.5), except for round cake (2.53) and cupcake (1.31), which demonstrated more favorable fatty acid balances. These results were consistent with the Nutritional Index, where round cake (9.19) and cupcake (2.86) exceeded the breast milk-based threshold for good fat quality, while chocochip biscuits (0.81) and banana bilayer cake (0.92) scored poorly. Although products like potato chips and mix nimko had relatively high desirable fatty acid levels (DFA = 27.89 and 20.34, respectively), their high content of hypercholesterolemic fatty acids (OFA > 11) undermined their overall healthiness.

The findings indicate that while the lipid indices of round cake and cupcake are relatively favorable, these products remain nutritionally inadequate due to their high sugar and refined carbohydrate content, which can drive rapid glucose spikes, de novo lipogenesis, and excess energy intake, coupled with low levels of essential micronutrients and dietary fiber. Collectively, these results underscore that high-fat snack products, despite some favorable fatty acid profiles, generally provide suboptimal nutrition for infants and young children. This highlights the urgent need to reformulate snacks by reducing harmful saturated fats and refined carbohydrates, while enhancing beneficial unsaturated fats and nutrient density, to ensure healthier dietary options for this vulnerable population.

### 3.4. Oxidative Stability of Oil Extracted from Snacks

Peroxide Value: Table 3 shows that the average values of peroxide for the samples of biscuit oil, cake, chips, and nimko did not exceed the recommended values. Further, the peroxide values for chips and nimko were comparably higher than other snack fat/oil samples, such as biscuits and cakes. Most of the international standards regarding the quality of frying oils cite limits for the peroxide value and free fatty acid percentage. The currently assessed samples did not exceed the regulated PV (>10) recommended by the Pakistan standard and quality control authority (PSQCA) and other agencies like European regulations and WHO/FAO [40,41,42,43]. Sulieman et al. (2006) [44] stated that a good quality vegetable oil for frying should have a peroxide index of <2 mEq O_2_/kg. The present study shows a wide range of peroxide values, varying from 0.16 ± 0.1 to 4.133 ± 0.4 mEq O_2_/kg. However, values are within the permissible limit set by various regulating agencies but higher than 2 mEq O_2_/kg, which suggests that some oils are not of good quality.

Free fatty acids: Free fatty acids were determined, ranging from 0.13 ± 0.03 to 2.89 ± 0.04%. Higher values in some samples of oils (for example, cooking oil in restaurants) revealed hydrolytic degradation of triacyl glycerides [45,46]. This is because the release of water from food towards triacyl glycerides in the fatty acid medium is connected to hydrolytic degradation in frying processes. Acidity can be determined with the help of the moisture of each sample item, as well as the frying oil’s processing parameters. As a result, it is recommended that frying oil be discarded occasionally rather than by adding more into it, as the oil is absorbed by the fried products. This treatment would slow or stop the rate of hydrolytic changes, concealing or delaying the deterioration. Nevertheless, frying oils should not have a free fatty acid content greater than 1%, specifically since free fatty acids are removed during the industrial refining process. The higher free fatty acid content was evaluated in 13% of snack samples, including rusk cake, which has 2.89%, and fruit cake, with a 1.1% FFA value. Besides dal moth, nimko the values were 2.27%, which is above the standard value. On the contrary, FFA and peroxide values for biscuits were found to be within the limits in a report from Argentina [47]. This may be due to food control systems are in practice in the country.

Conjugated diene: The values of conjugated diene for biscuits varied from 4.01 to 14.9; for cakes, the value varied from 2.43 to 10.5; for chips, it ranged from 0.697 to 2.17; and for nimko, it ranged from 4.84 to 7.61 (Table 3). From the above data, it was observed that the value of conjugated diene was higher in biscuits, which shows that the oil of biscuits is oxidized. On the other hand, absorbance measurements at 232 nm (conjugated diene) and 272 nm (conjugated triene) are used to determine fat oxidation, with parameter values varying depending on oxidation conditions [48]. CD values > 4 correlate with unacceptable peroxide index values (i.e., <10 mEq O_2_/kg in Europe). In the current study, the values of conjugated diene range from 3.36 ± 1.9 to 14.86 ± 0.67. The values of CD are higher in 77% of biscuits, 66% of cakes, 100% of chips, and 100% of nimko.

Conjugated triene: The values of conjugated triene are given in Table 3. It was observed from the above data that the value for biscuits ranged from 0.705 to 1.28; for cake, the value ranged from 0.858 to 3.83; for chips, this ranged from 0.697 to 2.17; and for nimko, this varied from 1.71 to 2.05. The CT for chips was higher compared to the other snacks under study, probably due to the high temperature and frying time of oil that cause the change from single bonds to double bonds, respectively. In turn, K270 is associated with the representation of conjugated trienes. According to Lopez-Varela et al., CT values increase in frying vegetable oils, and CT values exceeded the seven-unit limit [49]. In our study, the values ranged from 0.70 ± 0.01 to 1.27 ± 0.08, and it was observed that the values are within the allowable limit.

P-anisidine value: Table 3 reveals that the value of p-AnV for biscuits ranged from 6.23 to 23.42; for cakes, this ranged from 8.57 to 41.1; for chips, it varied from 12.23 to 30.14; and for nimko, it ranged from 7.29 to 16.62. By comparing the above data, it was observed that the p-AnV was higher in cakes, and it was responsible for an unpleasant flavor and unacceptable food quality. As expected, the photometric measurement of p-AnV at 350 nm wavelengths was relatively low for all oil samples. The p-AnV gives an estimation of secondary oxidation products such as 2-alkanals, 2,4-dienals, and unsaturated aldehydes. According to the literature, good-quality oils should have a p-AnV < 10 [50,51]. Index values for the present study ranged from 6.23 ± 0.58 to 40.72 ± 1.23. It was observed that the value of p-AnV is higher in 66% of biscuits, 83% of cakes, 100% of chips, and 33% of nimko oil, consisting of chocolate sandwich biscuits, cream sandwich biscuits, sugar-coated biscuits, zera biscuits, crackers, whole wheat biscuits fruit cakes, banana layered cake, round cake, rusk cake, cupcakes, dal moth nimko, and all samples of chips.

Total oxidation Index: The TOTOX index is used to investigate the total deterioration of fats and oils, relates the peroxide values to the p-AnV, and gives qualitative or quantitative information about the primary and secondary oxidation products. Table 3 indicates the total oxidation index values that are calculated from peroxide and P-Av values. It was observed from the above data for biscuits that the value varied from 7.47 to 29.4; for cakes, it ranged from 8.57 to 41.1; for chips, it varied from 14.76 to 38.4; for nimko, it varied from 10.31 to 19.92. The total oxidation was higher in cakes, which may be due to the high baking temperature of the oil. It affects sensory data like color, order, and flavor, which is responsible for the formation of several oxidation products [52]. Generally, the recommended TOTOX value is less than or equal to 19.5 mEq kg^−1^ as reported in the literature [53]. The analyzed samples presented a wide range of total values, ranging from 7.15 ± 0.61 to 41.72 ± 1.36. The total oxidation index values are higher in biscuits (22%), cakes (33%), chips (5%), and nimko (33%), including chocolate sandwich biscuits, whole wheat biscuits, banana layered cakes, cupcakes, potato chips, corn flour chips, wheat flour and potato starch chips, and mix nimko.

## 4. Conclusions

The findings of this study reveal that children aged 6–24 months in rural Matiari, Pakistan, commonly consume high-fat snacks such as biscuits, cakes, chips, and nimko. These products contain high levels of saturated fats, highlight the presence of trans fats, and are characterized as energy-dense and nutritionally imbalanced. Cakes are low in fat compared to other snacks, but due to the high presence of sugar and carbohydrates and low levels of micronutrients, they are therefore not counted as a nutritious or healthy food choices, and their frequent consumption, particularly among infant children, raises significant concerns about long-term health risks, including diet-related non-communicable diseases (NCDs). Given their widespread availability and low cost, such snacks are often preferred over healthier options. However, healthier alternatives, such as fruit, can be similarly affordable if families are guided to make informed food choices.

There is a pressing need for multi-sectoral action involving policymakers, food inspectors, public health professionals, and educators to regulate the nutritional quality of snack foods, improve their formulation using standard protocols, enhance labeling and advertising standards, and translate our findings into national dietary guidelines and public health policies targeting infant and toddler nutrition in Pakistan and similar low- and middle-income countries while promoting community-based nutrition education to foster healthier dietary habits from an early age.

## 5. Limitations of Study

A key limitation of this study is that trans fats were not quantitatively analyzed due to limited resources. In addition, about 50% of the planned samples could not be collected for analysis due to differences in sampling and data collection periods. Another constraint was the inability to assess the oxidative stability of the snack products over different storage durations. This means we could not evaluate how fat quality might deteriorate over time or its potential impact on health and product safety. These limitations point to the need for future research with a wider range of samples and time-based analyses to better understand the composition and stability of fats in commonly consumed high-fat snacks.

## Figures and Tables

**Table 1:** Fatty acid composition (g/100 g) of high-fat snacks consumed by children aged 6–24 months.

Samples	Lauric Acid C12:0	Myristic Acid C14:0	Palmitic Acid C16:0	Stearic Acid C18:0	Arachidic Acid C20:0	Palmitoleic Acid C16:1 (n-9)	Oleic Acid C18:1 (n-9)	Linoleic Acid C18:2n 9c,12c	ΣSFA	ΣUFA	ΣMUFA	ΣPUFA	UFA/SFA
Chocolate Sandwich Biscuits	_	0.18 ± 0.01	9.76 ± 0.1	1.55 ± 0	0.09 ± 0	_	12.26 ± 0.1	1.97 ± 0	11.58	14.23	12.26	1.97	1.23
Cream Sandwich Biscuits	0.49 ± 0	0.4 ± 0.01	10.76 ± 0.9	1.66 ± 0.4	0.1 ± 0.1	_	13.34 ± 0.4	1.96 ± 0	13.41	15.30	13.34	1.96	1.14
Sugar-Coated Biscuits	_	0.12 ± 0	6.5 ± 2.2	1.35 ± 2.7	0.07 ± 0	_	7.5 ± 3.6	1.11 ± 1.3	8.04	8.61	7.50	1.11	1.07
Wafer	_	0.22 ± 0	8.98 ± 3.8	1.33 ± 0.3	0.09 ± 0	_	16.47 ± 4.2	0.6 ± 0.1	10.62	17.07	16.47	0.60	1.61
Zera Biscuits	_	0.13 ± 0	6.91 ± 0.5	1.15 ± 0.4	0.09 ± 0.1	_	9.16 ± 0	1.47 ± 0.1	8.28	10.63	9.16	1.47	1.28
Whole Wheat Biscuits	_	0.1 ± 0	5.3 ± 0.2	0.82 ± 0.1	0.06 ± 0	_	7.03 ± 0.4	1.13 ± 0.1	6.28	8.16	7.03	1.13	1.30
Peanut Biscuits	_	0.14 ± 0	7.91 ± 0	1.2 ± 0	0.16 ± 0	_	11.5 ± 0	2.46 ± 0	9.41	13.96	11.50	2.46	1.48
Chocochip Biscuits	2.27 ± 0	1.25 ± 0.1	11.55 ± 0.9	2.01 ± 0.3	_	_	10.18 ± 0.3	1.86 ± 0.1	17.08	12.04	10.18	1.86	0.70
Crackers	_	0.07 ± 0.1	3.91 ± 0.3	0.59 ± 0.7	0.04 ± 0.1	_	4.63 ± 0.2	0.7 ± 0.3	4.61	5.33	4.63	0.70	1.16
Fruit Cake	_	0.11 ± 0	5.57 ± 0.7	0.82 ± 0.3	0.05 ± 0	_	6.93 ± 0.4	1.63 ± 0.1	6.55	8.56	6.93	1.63	1.31
Sponge Cake	_	0.12 ± 0.1	5.79 ± 3.2	1.08 ± 0.6	0.06 ± 0	0.06 ± 0	6.74 ± 4.6	1.15 ± 0.7	7.05	7.95	6.80	1.15	1.13
Round Cake	_	0.07 ± _-	0.69 ± 0.22	0.29 ± 0.1	0.06 ± 0	0.06 ± 0.1	7.29 ± 1.1	1.74 ± 0.6	1.11	9.09	7.35	1.74	8.19
Banana Bilayer Cake	_	0.11 ± 0.3	6.13 ± 0.6	2.09 ± 2.8	_	_	6.02 ± 3.6	1.61 ± 0.1	8.33	7.63	6.02	1.61	0.92
Rusk Cake	_	0.17 ± 0	7.79 ± 0.3	1.31 ± 0.8	_	_	9.63 ± 1.3	1.51 ± 0.2	9.27	11.14	9.63	1.51	1.20
Cup Cake	_	0.05 ± 0	3.04 ± 0	0.82 ± 0	0.06 ± 0	_	7.1 ± 0	4.05 ± 0	3.97	11.15	7.10	4.05	2.81
Potato Chips	_	0.32 ± 0	16.61 ± 0.1	2.26 ± 0.1	0.3 ± 0	_	21.07 ± 0.3	4.56 ± 0.5	19.49	25.63	21.07	4.56	1.32
Wheat Flour Chips	0.03 ± 0	0.07 ± 0	3.15 ± 0	0.46 ± 0	0.05 ± 0	_	4.98 ± 0	_	3.76	4.98	4.98	0.00	1.32
Corn Flour Chips	_	0.08 ± 0.1	4.46 ± 0.3	0.59 ± 0.1	0.06 ± 0.1	_	5.56 ± 1.5	1.09 ± 1.5	5.19	6.65	5.56	1.09	1.28
Wheat Flour and Potato Starch	_	0.06 ± 0	3.06 ± 0	0.46 ± 0	0.06 ± 0	_	5.08 ± 0	_	3.64	5.08	5.08	0.00	1.40
Mix Nimko	_	0.23 ± 0	12.44 ± 0.3	1.62 ± 0.1	0.13 ± 0	_	15.31 ± 0.2	3.41 ± 0.2	14.42	18.72	15.31	3.41	1.30
Dal Moth Nimko	_	0.22 ± 0	11.91 ± 0.2	1.59 ± 0	0.18 ± 0.2	_	14.74 ± 1.5	2.72 ± 1.4	13.90	17.46	14.74	2.72	1.26
Mix Nimko Local Bakery	_	0.22 ± 0	11.3 ± 0.8	1.64 ± 0.3	0.13 ± 0	_	17.54 ± 1.2	_	13.29	17.54	17.54	0.00	1.32

**Table 2:** Lipid quality indices of high-fat snacks consumed under the age of 6–24 months.

Samples	AI	TI	h/H	NI	DFA	OFA	Lipid Quality Interpretation with Breast Milk
Chocolate Sandwich Biscuits	0.7	0.81	0.2	1.24	15.8	9.94	AI/TI high, h/H very low → Worse than breast milk; poor lipid balance for children
Cream Sandwich Biscuits	0.76	0.84	0.17	1.19	17	11.7	High AI/TI, very low h/H → Poor for pediatric intake
Sugar-Coated Biscuits	0.77	0.92	0.17	1.08	9.96	6.62	High AI/TI, low h/H → Suboptimal for children; DFA slightly lower than breast milk
Wafer	0.54	0.62	0.07	1.62	18.4	9.2	Moderate AI/TI, very low h/H → Worse than breast milk; not ideal for children
Zera Biscuits	0.66	0.77	0.21	1.3	11.8	7.04	AI/TI above breast milk, h/H low → Needs improvement
Whole Wheat Biscuits	0.66	0.76	0.21	1.31	8.98	5.4	AI/TI high, h/H low → Suboptimal fat quality for children
Peanut Biscuits	0.58	0.66	0.3	1.51	15.2	8.05	Closer to breast milk h/H, DFA moderate → Acceptable for children
Chocochip Biscuits	1.25	1.23	0.12	0.81	14.1	15.1	Very high AI/TI, very low h/H → Poor fat profile; highly unsuitable
Crackers	0.75	0.86	0.18	1.17	5.92	3.98	AI/TI high, h/H very low → Poor lipid quality vs. breast milk
Fruit Cake	0.66	0.76	0.29	1.32	9.38	5.68	AI/TI high, h/H low → Moderate to poor relative to breast milk
Sponge Cake	0.75	0.89	0.2	1.13	9.03	5.91	High AI/TI, low h/H → Poor lipid quality for children
Round Cake	0.08	0.11	2.53	9.19	9.38	0.76	AI/TI much lower, h/H excellent → Exceeds breast milk quality; very healthy for children
Banana Bilayer Cake	0.82	1.09	0.26	0.92	9.72	6.24	AI/TI high, h/H low → Poor relative to breast milk
Rusk Cake	0.71	0.83	0.19	1.2	12.5	7.96	High AI/TI, low h/H → Suboptimal for children
Cup Cake	0.28	0.35	1.31	2.86	12	3.09	AI/TI slightly lower, h/H moderate → Acceptable, closer to breast milk profile
Potato Chips	0.66	0.75	0.27	1.34	27.9	16.9	AI/TI high, OFA very high → Energy-dense but worse than breast milk; moderate DHA/PUFA not accounted for
Wheat Flour Chips	0.65	0.74	0	1.35	5.44	3.25	AI/TI high, h/H very low → Poor fat quality vs. breast milk
Corn Flour Chips	0.68	0.77	0.24	1.29	7.24	4.54	Moderate AI/TI, h/H low → Below breast milk standards
Wheat + Potato Starch Chips	0.61	0.71	0	1.42	5.54	3.12	AI/TI high, h/H very low → Poor lipid profile for pediatric intake
Mix Nimko	0.68	0.76	0.27	1.31	20.3	12.7	AI/TI slightly above breast milk, h/H lower → Average nutritional quality
Dal Moth Nimko	0.69	0.79	0.22	1.27	19.1	12.1	AI/TI above breast milk, h/H low → Borderline acceptable fat quality
Local Bakery Mix Nimko	0.66	0.75	0	1.33	19.2	11.5	AI/TI high, h/H very low → Needs improvement for children

**Table 3:** The oxidative stability parameters of oil extracted from high-fat snacks.

Samples	*p*-Anisidine Value (Arbitrary Unit)	Peroxide Value (mEq/kg)	TOTOX (Arbitrary Unit)	Conjugated Diene $\left(E_{1cm}^{1\%}\right)$	Conjugated Triene $\left(E_{1cm}^{1\%}\right)$	FFA (%)
Chocolate sandwich biscuits	28.9 ± 3.3	0.26 ± 0.1	29.4 ± 3.1	4.0 ± 1.2	1.3 ± 0.1	0.22 ± 0.03
Cream sandwich biscuits	11.2 ± 2.9	0.4 ± 0.2	11.9 ± 2.8	8.3 ± 0.4	1.1 ± 0.2	0.27 ± 0.03
Sugar-coated biscuits	18.4 ± 1.7	0.5 ± 0.1	19.4 ± 2.2	5.9 ± 1.2	0.9 ± 0.1	0.28 ± 0.02
Wafer	6.2 ± 0.6	0.5 ± 0.1	7.2 ± 0.61	10.5 ± 3.7	0.9 ± 0.2	0.13 ± 0.03
Zera biscuits	12.9 ± 0.5	0.4 ± 0.2	13.8 ± 0.87	5.6 ± 0.1	1.13 ± 0.1	0.15 ± 0.02
Whole wheat biscuits	23.4 ± 2.9	1.2 ± 0.1	25.9 ± 1.7	3.4 ± 1.9	0.9 ± 0.2	0.21 ± 0.07
Peanut biscuits	10.4 ± 1.5	1.7 ± 0.2	13.7 ± 1.8	14.8 ± 0.7	1.3 ± 0.1	0.25 ± 0.02
Chocochip biscuits	9.7 ± 0.6	0.3 ± 0.1	10.4 ± 0.7	6.8 ± 2.3	1.1 ± 0.2	0.30 ± 0.03
Crackers	11.9 ± 4.0	0.3 ± 0.1	12.5 ± 3.8	6.5 ± 0.2	0.70 ± 0.01	0.17 ± 0.03
Fruit cake	13.5 ± 1.5	0.9 ± 0.2	15.3 ± 1.9	4.6 ± 1.0	3.8 ± 0.04	1.11 ± 0.2
Sponge cake	6.0 ± 1.7	1.6 ± 0.2	9.2 ± 1.9	6.7 ± 0.6	0.95 ± 0.1	0.17 ± 0.05
Round cake	18.4 ± 3.6	0.4 ± 0.2	19.3 ± 3.4	2.4 ± 0.5	0.85 ± 0.04	0.37 ± 0.12
Banana bilayer cake	23.9 ± 1.1	0.4 ± 0.2	24.8 ± 0.7	10.5 ± 0.3	1.9 ± 0.01	1.02 ± 0.17
Rusk cake	18.4 ± 0.8	0.5 ± 0.1	19.4 ± 0.9	6.1 ± 1.9	0.9 ± 0.1	2.89 ± 0.04
Cup cake	40.7 ± 1.2	0.5 ± 0.1	41.7 ± 1.4	10.2 ± 3.4	3.8 ± 0.1	1.03 ± 0.30
Potato chips	13.0 ± 0.7	3.8 ± 0.3	20.6 ± 1.4	6.5 ± 3.0	1.67 ± 0.2	0.26 ± 0.03
Wheat flour chips	12.2 ± 2.1	1.3 ± 0.2	14.8 ± 2.1	5.8 ± 3.2	0.85 ± 0.02	0.58 ± 0.07
Corn flour chips	17.5 ± 4.6	1.4 ± 0.2	20.3 ± 4.2	9.9 ± 4.3	0.7 ± 0.1	0.62 ± 0.05
Wheat flour and potato starch	30.1 ± 0.1	4.1 ± 0.4	38.4 ± 0.9	13.7 ± 0.3	2.2 ± 0.1	0.58 ± 0.1
Mix nimko	7.3 ± 0.1	3.3 ± 0.6	13.9 ± 1.1	7.60 ± 0.32	2.04 ± 0.04	0.99 ± 0.26
Dal moth nimko	3.6 ± 3.4	3.4 ± 0.6	10.3 ± 3.2	5.05 ± 0.65	2.04 ± 0.10	2.27 ± 0.24

## Data Availability

The original contributions presented in the study are included in the article. Further inquiries can be directed to the corresponding authors.

## References

[1] Wagner K.-H., Plasser E., Proell C., Kanzler S. (2008). Comprehensive studies on the trans fatty acid content of Austrian foods: Convenience products, fast food and fats. Food Chem..

[2] UNICEF The State of the World’s Children 2023: For Every Child, Vaccination. UNICEF Innocenti—Global Office of Research and Foresight, Florence, April 2023. https://www.unicef.org/reports/state-worlds-children-2023.

[3] Black R.E., Victora C.g., Walker S.P., Bhutta Z.A., Christian P., de Onis M., Ezzati M., Grantham-McGregor S., Katz J., Martorell R. (2013). Maternal and child undernutrition and overweight in low-income and middle-income countries. Lancet.

[4] Victora C.G., Adair L., Fall C., Hallal P.C., Martorell R., Richter L., Sachdev H.S., Maternal and Child Undernutrition Study Group (2008). Maternal and child undernutrition: Consequences for adult health and human capital. Lancet.

[5] Georgieff M.K., Ramel S.E., Cusick S.E. (2018). Nutritional influences on brain development. Acta Paediatr..

[6] Birch L.L., Ventura A.K. (2009). Preventing childhood obesity: What works?. Int. J. Obes..

[7] Paul I.M., Bartok C.J., Downs D.S., Stifter C.A., Ventura A.K., Birch L.L. (2009). Opportunities for the primary prevention of obesity during infancy. Adv. Pediatr..

[8] Bajwa M.J., Nadeem M.A., Khalid N. (2023). Educating Parents on Children Daily Dietary Intakes Concentration of Heavy Metal in Biscuits and Rusks in Pakistan. Pak. Soc. Sci. Rev..

[9] Nestel P.J., Mori T.A. (2022). Dairy foods: Is its cardiovascular risk profile changing?. Curr. Atheroscler. Rep..

[10] Nestel P. (2014). Trans fatty acids: Are its cardiovascular risks fully appreciated?. Clin. Ther..

[11] Soomro S.I., Memon N., Bhanger M.I., Memon S., Memon A.A. (2016). Mineral content of Pakistani foods: An update of food composition database of Pakistan through indirect method. J. Food Compos. Anal..

[12] Clark H., Coll-Seck A.M., Banerjee A., Peterson S., Dalglish S.L., Ameratunga S., Balabanova D., Bhan M.K., Bhutta Z.A., Borrazzo A. (2020). A future for the world’s children? A WHO–UNICEF–Lancet Commission. Lancet.

[13] Leonez D.G.V.R., Melhem A.R.d.F., Vieira D.G., de Mello D.F., Saldan P.C. (2020). Complementary feeding indicators for children aged 6 to 23 months according to breastfeeding status. Rev. Paul. Pediatr..

[14] Soomro S.I., Jamil Z., Memon N., Ahmed S., Umrani F., Choudhri I.A., Mohammed S., Qureshi K., Raza G., Jakhro S. (2024). Nutrient dataset development via FAO/INFOODS approach for infant nutritional survey in rural Matiari, Pakistan. J. Food Compos. Anal..

[15] FAO/INFOODS (2012). Guidelines for Food Matching.

[16] Puwastien P., Siong T.E., Kantasubrata J., Craven G., Feliciano R.R., Judprasong K. (2011). Asean Manual of Nutrient Analysis.

[17] Sayre D.A. (2014). INSDE ISO 14000: The Competitive Advantage of Environmental Management.

[18] Paszczyk B., Łuczyńska J. (2020). The comparison of fatty acid composition and lipid quality indices in hard cow, sheep, and goat cheeses. Foods.

[19] Ulbricht T., Southgate D. (1991). Coronary heart disease: Seven dietary factors. Lancet.

[20] Crowe T.D., White P.J. (2001). Adaptation of the AOCS official method for measuring hydroperoxides from small-scale oil samples. J. Am. Oil Chem. Soc..

[21] AOCS (1960). Method Cd 8–53.

[22] Saldivar S. (2016). Snack Foods: Types and composition. Encyclopedia of Food and Health.

[23] Chester M.A. (1997). Nomenclature of glycolipids (IUPAC recommendations 1997). Pure Appl. Chem..

[24] Saini D., Barthwal R., Sharma S.K., Rawat N. (2022). Chemistry of Food Fats, Oils, and Other Lipids.

[25] National Academies of Sciences, Engineering, and Medicine (2005). Dietary Reference Intakes for Energy, Carbohydrate, Fiber, Fat, Fatty Acids, Cholesterol, Protein, and Amino Acids.

[26] Hunter J.E. (2006). Dietary trans fatty acids: Review of recent human studies and food industry responses. Lipids.

[27] Geraldo J.M., Alfenas R.D.C. (2008). Role of diet on chronic inflammation prevention and control: Current evidence. Arq. Bras. Endocrinol. Metabol..

[28] Havlicekova Z., Jesenak M., Banovcin P., Kuchta M. (2015). Beta-palmitate—A natural component of human milk in supplemental milk formulas. Nutr. J..

[29] Lopes C., Aro A., Azevedo A., Ramos E., Barros H. (2007). Intake and adipose tissue composition of fatty acids and risk of myocardial infarction in a male Portuguese community sample. J. Am. Diet. Assoc..

[30] Vardavas C., Yiannopoulos S., Kiriakakis M., Poulli E., Kafatos A. (2007). Fatty acid and salt contents of snacks in the Cretan and Cypriot market: A child and adolescent dietary hazard. Food Chem..

[31] Simopoulos A.P. (1999). Essential fatty acids in health and chronic disease. Am. J. Clin. Nutr..

[32] Joshee K., Abhang T., Kulkarni R. (2019). Fatty acid profiling of 75 Indian snack samples highlights overall low trans fatty acid content with high polyunsaturated fatty acid content in some samples. PLoS ONE.

[33] Teixeira B.C., Lopes A.L., Macedo R.C.O., Correa C.S., Ramis T.R., Ribeiro J.L., Reischak-Oliveira A. (2014). Inflammatory markers, endothelial function and cardiovascular risk. J. Vasc. Bras..

[34] Aftab Kandhro A.K., Sherazi S.T.H., Mahesar S.A., Bhanger M.I., Talpur M.Y., Arain S. (2008). Monitoring of fat content, free fatty acid and fatty acid profile including trans-fat in Pakistani biscuits. J. Am. Oil Chem. Soc..

[35] Moss J. (2006). Labeling of trans fatty acid content in food, regulations and limits—The FDA view. Atheroscler. Suppl..

[36] Van Poppel G., van Erp-Baart M.-A., Leth T., Gevers E., Van Amelsvoort J., Lanzmann-Petithory D., Kafatos A., Aro A. (1998). Trans fatty acids in foods in Europe: The TRANSFAIR study. J. Food Compos. Anal..

[37] Roe M., Pinchen H., Church S., Elahi S., Walker M., Farron-Wilson M., Buttriss J., Finglas P. (2013). Trans fatty acids in a range of UK processed foods. Food Chem..

[38] Krettek A., Thorpenberg S., Bondjers G. (2008). Trans Fatty Acids and Health: A Review of Health Hazards and Existing Legislation.

[39] Assaf R.R. (2014). Overview of local, state, and national government legislation restricting trans fats. Clin. Ther..

[40] Maingrette F., Li L., Renier G. (2008). C-reactive protein enhances macrophage lipoprotein lipase expression. J. Lipid Res..

[41] Pakistan Standard Quality Control Authority (PSQCA) (2023). Pakistan Standard Specification for Cooking Oil and Banaspati (PS:2858–2023).

[42] Codex Alimentarius Commission (1999). Codex Standard for Named Vegetable Oils (CODEX-STAN 210–1999).

[43] FAO/WHO (1994). Fats and Oils in Human Nutrition: Report of a Joint Expert Consultation.

[44] Sulieman A.E.R.M., El-Makhzangy A., Ramadan M.F. (2006). Antiradical performance and physicochemical characteristics of vegetable oils upon frying of French fries: A preliminary comparative study. J. Food Lipids.

[45] Peng G.-J., Chang M.-H., Fang M., Liao C.-D., Tsai C.-F., Tseng S.-H., Kao Y.-M., Chou H.-K., Cheng H.-F. (2017). Incidents of major food adulteration in Taiwan between 2011 and 2015. Food Control.

[46] Sebastian A., Ghazani S.M., Marangoni A.G. (2014). Quality and safety of frying oils used in restaurants. Food Res. Int..

[47] Patrignani M., Conforti P.A., Lupano C.E. (2015). Lipid oxidation in biscuits: Comparison of different lipid extraction methods. J. Food Meas. Charact..

[48] Bozdemir S., Güneşer O., Yilmaz E. (2015). Properties and stability of deep-fat fried chickpea products. Grasas Aceites.

[49] Lopez-Varela S., Sánchez-Muniz F.J., Garrido-Polonio C., Arroyo R., Cuesta C. (1995). Relationship between chemical and physical indexes and column and HPSE chromatography methods for evaluating frying oil. Z. Für Ernährungswissenschaft.

[50] López-Sobaler A.M., Aparicio A., Rubio J., Marcos V., Sanchidrián R., Santos S., Pérez-Farinós N., Dal-Re M.Á., Villar-Villalba C., Yusta-Boyo M.J. (2019). Adequacy of usual macronutrient intake and macronutrient distribution in children and adolescents in Spain: A National Dietary Survey on the Child and Adolescent Population, ENALIA 2013–2014. Eur. J. Nutr..

[51] Totani N., Yasaki N., Doi R., Hasegawa E. (2017). Active Cooling of Oil after Deep-frying. J. Oleo Sci..

[52] Jacobsen C. (2010). Understanding and reducing oxidative flavour deterioration in foods. Oxidation in Foods and Beverages and Antioxidant Applications.

[53] Esfarjani F., Khoshtinat K., Zargaraan A., Mohammadi-Nasrabadi F., Salmani Y., Saghafi Z., Hosseini H., Bahmaei M. (2019). Evaluating the rancidity and quality of discarded oils in fast food restaurants. Food Sci. Nutr..

